# COVIDApp as an Innovative Strategy for the Management and Follow-Up of COVID-19 Cases in Long-Term Care Facilities in Catalonia: Implementation Study

**DOI:** 10.2196/21163

**Published:** 2020-07-17

**Authors:** Patricia Echeverría, Miquel Angel Mas Bergas, Jordi Puig, Mar Isnard, Mireia Massot, Cristina Vedia, Ricardo Peiró, Yolanda Ordorica, Sara Pablo, María Ulldemolins, Mercé Iruela, Dolors Balart, José María Ruiz, Jordi Herms, Bonaventura Clotet Sala, Eugenia Negredo

**Affiliations:** 1 Infectious Diseases Department Hospital Universitari Germans Trias i Pujol Badalona Spain; 2 Lluita contra la Sida Foundation Hospital Universitari Germans Trias i Pujol Badalona Spain; 3 Direcció Clínica Territorial Metropolitana Nord Institut Català de la Salut Barcelona Spain; 4 Servei d'Atenció Primària Vallès Occidental Direcció Atenció Primària Metropolitana Nord Institut Català de la Salut Barcelona Spain; 5 Servei d'Atenció Primària Barcelonès Nord Maresme Direcció Atenció Primària Metropolitana Nord Institut Català de la Salut Barcelona Spain; 6 Servei d'Atenció Primària Vallès Oriental Direcció Atenció Primària Metropolitana Nord Institut Català de la Salut Barcelona Spain; 7 Direcció Atenció Primària Metropolitana Nord Institut Català de la Salut Barcelona Spain; 8 Lluita contra la Sida Foundation Digital Health Department Doole Health S.L. Badalona Spain; 9 AIDS Research Institute-IRSICAIXA Hospital Universitari Germans Trias i Pujol Universitat Autònoma de Barcelona Barcelona Spain; 10 Centre for Health and Social Care Research (CESS) Faculty of Medicine University of Vic - Central University of Catalonia Barcelona Spain

**Keywords:** COVID-19, mobile health, app, COVIDApp, long-term care facilities, institutionalized individuals, mHealth, elderly, long-term, care, public health, management, surveillance

## Abstract

**Background:**

The coronavirus disease (COVID-19) pandemic has caused an unprecedented worldwide public health crisis that requires new management approaches. COVIDApp is a mobile app that was adapted for the management of institutionalized individuals in long-term care facilities.

**Objective:**

The aim of this paper is to report the implementation of this innovative tool for the management of long-term care facility residents as a high-risk population, specifically for early identification and self-isolation of suspected cases, remote monitoring of mild cases, and real-time monitoring of the progression of the infection.

**Methods:**

COVIDApp was implemented in 196 care centers in collaboration with 64 primary care teams. The following parameters of COVID-19 were reported daily: signs/symptoms; diagnosis by reverse transcriptase–polymerase chain reaction; absence of symptoms for ≥14 days; total deaths; and number of health care workers isolated with suspected COVID-19. The number of at-risk centers was also described.

**Results:**

Data were recorded from 10,347 institutionalized individuals and up to 4000 health care workers between April 1 and 30, 2020. A rapid increase in suspected cases was seen until day 6 but decreased during the last two weeks (from 1084 to 282 cases). The number of confirmed cases increased from 419 (day 6) to 1293 (day 22) and remained stable during the last week. Of the 10,347 institutionalized individuals, 5,090 (49,2%) remained asymptomatic for ≥14 days. A total of 854/10,347 deaths (8.3%) were reported; 383 of these deaths (44.8%) were suspected/confirmed cases. The number of isolated health care workers remained high over the 30 days, while the number of suspected cases decreased during the last 2 weeks. The number of high-risk long-term care facilities decreased from 19/196 (9.5%) to 3/196 (1.5%).

**Conclusions:**

COVIDApp can help clinicians rapidly detect and remotely monitor suspected and confirmed cases of COVID-19 among institutionalized individuals, thus limiting the risk of spreading the virus. The platform shows the progression of infection in real time and can aid in designing new monitoring strategies.

## Introduction

The disease caused by severe acute respiratory syndrome coronavirus 2 (SARS-CoV-2), called coronavirus disease (COVID-19), was initially identified in December 2019 as a case of pneumonia in Wuhan, China [1,2]. COVID-19 has since become a global pandemic that is affecting more than 200 countries worldwide, with more than 3.5 million people infected globally and more than 240,000 related deaths as of April 30, 2020 [3]. The World Health Organization declared COVID-19 a pandemic on March 11, 2020, and called for coordinated mechanisms to provide a response to the infection across various health sectors [4]. On March 14, the Spanish authorities declared the pandemic to be a national emergency [5].

The rapid spread of the infection and its severity in a considerable percentage of patients has necessitated unprecedented public health measures. Health systems worldwide are working against the clock and taking exceptional measures to address the crisis. Health professionals require methods to detect, treat, and monitor patients with COVID-19 effectively and efficiently and to prevent further transmission of the disease.

The health crisis generated by COVID-19 requires new approaches to disease management, especially in the case of older individuals, as this population is especially vulnerable to severe illnesses and early data point to higher mortality from COVID-19 in this population than in young and middle-aged patients [6,7]. In addition, the high risk of transmission of COVID-19 in long-term care facilities (nursing homes and other institutions) with vulnerable populations and the resulting challenge of controlling the epidemic in these settings have necessitated innovative responses [8,9]. In this sense, expert recommendations indicate that medical decisions should include rapid screening to identify suspected cases early and to facilitate on-site management or transfer to hospital, as applicable [10].

Given this scenario, we adapted a mobile health app that was designed in 2015 [11] and that has since been used for the clinical management of HIV-infected persons in our HIV Unit (+Approp). For the last two years, the app has been used in additional scenarios, such as clinical management of the general population and of patients with chronic conditions (Doole Health). COVIDApp is an adapted version of this app that aims to address the current COVID-19 crisis by closely monitoring institutionalized subjects and their contacts through providing remote medical attention. The objective of this paper is to report the use of this innovative tool for the management of long-term care facility residents as a high-risk population, specifically for early identification and self-isolation of suspected cases, remote monitoring of mild cases, and real-time monitoring of the progression of the infection.

## Methods

### Study Design, Objectives, and Population

We describe the implementation of a mobile app (COVIDApp) for the management of COVID-19 in institutionalized persons in long-term care facilities (older residents and individuals with physical and mental disabilities). This innovative strategy addresses the COVID-19 pandemic by intervening in prevention, care, and epidemiology.

The COVIDApp tool was optimized to meet the following objectives: early identification and self-isolation of persons suspected of having COVID-19 for rapid diagnosis of positive cases by real-time reverse transcriptase–polymerase chain reaction (RT-PCR), thus minimizing the risk of transmission in long-term care facilities; remote treatment and monitoring of mild cases of COVID-19 self-isolating at nursing homes when indicated; and real-time monitoring of the progression of the infection and its consequences in these at-risk facilities.

A total of 196 care centers (169 nursing homes and 27 institutions for people with physical and mental disabilities) participated in collaboration with 64 primary care teams from the northern area of Barcelona, Catalonia (Barcelonès Nord, Maresme, Vallès Oriental, and Vallès Occidental Valles), which has a reference population of 1,986,032 inhabitants. In Catalonia, the entire population is covered by publicly financed health services, and universal care is provided by primary care teams and hospitals. Regarding long-term care facilities, although some of these facilities are private, all citizens are covered by public health services. For that reason, each long-term care facility has a primary care team of reference.

We began using COVIDApp as a support tool for the clinical response of primary care teams to the epidemiological crisis on April 1, 2020. The data reported in this paper were registered on the platform between April 1 and 30, 2020.

### Endpoints

The parameters reported by health care staff at each institution with respect to all residents and caregivers were the number of persons with signs and/or symptoms of COVID-19 (suspected/symptomatic cases), number of persons with a diagnosis of SARS-CoV-2 by RT-PCR, number of residents remaining asymptomatic for more than 14 days, total number of deaths and deaths in suspected cases, number of suspected cases in health care workers, and number of isolated health care workers (confirmed cases, suspected cases, or contacts).

The number of high-risk facilities was also described. We categorized a long-term care facility as a high-risk center if it presented one or more of the following risk factors for two or more consecutive days: reporting by long-term care facility managers of difficulties managing the crisis (requiring action from local or regional authorities), reduced number of available health care professionals due to suspected or confirmed infection, lack of personal protective equipment (PPE) or need to disinfect the area, and situations where primary care teams detected that long-term care facility staff experienced difficulties complying with clinical recommendations or understanding epidemiological recommendations for prevention of new infections.

### COVIDApp Functions

COVIDApp is an easily accessible mobile health app that is available in the Google Play Store for the Android platform and in the Apple Store in iOS format; the app facilitates direct communication between long-term care facilities and primary care teams. Health personnel can access the app from any computer through a webpage. However, only authorized personnel can access the back office of the app to upload patients’ information.

COVIDApp provides information on facility residents in real time, including vital signs (eg, temperature, heart and respiratory rate, blood pressure, and oxygen saturation rate) and symptoms (eg, cough, expectoration, dyspnea, vomiting, diarrhea, or confusion). The platform provides a daily report of the numbers of suspected or confirmed COVID-19 cases, isolated cases, people remaining asymptomatic for more than 14 days, and deaths. COVIDApp also enables communication via chat or video between the health care team and the patient’s family and can be used to send different types of messages (eg, recommendations or treatment protocols), although this tool has not yet been activated. The app is implemented using redundant servers, periodic and encrypted backups, information encrypted via transport layer security (TLS) and HTTPS, and an Amazon Web Services global cloud infrastructure.

COVIDApp functions in various stages. First, vital signs and symptoms from all suspected cases are monitored daily at an individualized frequency (1 to 3 times per day) by health personnel at the institutions and are collected in the platform in real time. An immediate alert is sent to the primary care team through activation of an alarm via the app when people develop signs or symptoms related to COVID-19. Second, following an alarm, a clinical assessment by the primary care team is planned within 12 to 24 hours. Third, after the initial assessment, several measures are recommended, as follows: preventive epidemiological recommendations such as compartmentalization of specific areas and isolation of suspected cases and contacts; measures for staff to prevent infection, including PPE; RT-PCR testing; and reassessment of isolation measures based on test results. Fourth, suspected cases are isolated until the RT-PCR test result is available (within 24 hours), and cases who test positive for SARS-CoV-2 remain isolated and quarantined, receive appropriate treatment, and are monitored twice daily. Fifth, clinical progress (vital signs, symptoms, and clinical opinion) is reported daily by the long-term care facility staff via the app. Finally, clinical treatment is provided based on an individualized care plan: mild cases receive acute and supportive treatment, severe cases are transferred to hospital, and more severe cases may receive end-of-life palliative care. All patients remain in the center, except for severe cases, who are transferred to hospital.

## Results

During 30 days of follow-up using the platform, we managed data from more than 10,000 institutionalized individuals and up to 4000 health care workers. These data are a key element of the project and are shown in Table 1. The table shows the number of residents along with the percentages of centers that reported data on the platform each week. Because the numbers varied over the 30 days depending on the mobility of some residents, the number of deaths, and the number of centers reporting data daily on the platform, we present the data available at the end of each week throughout the 30-day period. The percentage of the 196 institutions that reported data was very high and increased over time, from 174 (88.8%) at day 9 to 190 (96.9%) at day 30.

**Figure 1:**
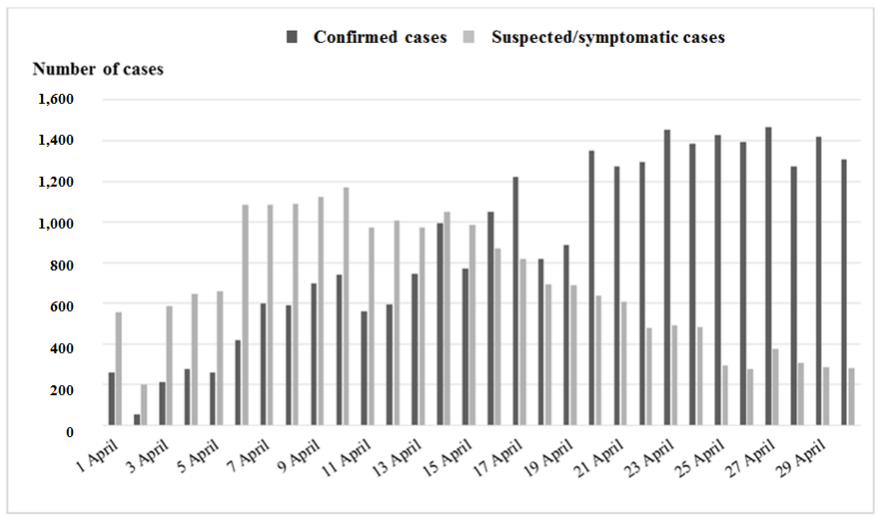
Numbers of suspected/symptomatic cases and confirmed cases of coronavirus disease among residents as reported by long-term care facility health care staff through COVIDApp over 30 days.

Figure 1 shows the information provided by long-term care facility staff on suspected/symptomatic and confirmed COVID-19 cases over time. A rapid increase in the number of suspected cases was seen until day 6; this number remained stable until day 14 and decreased during the last 2 weeks.

**Figure 2:**
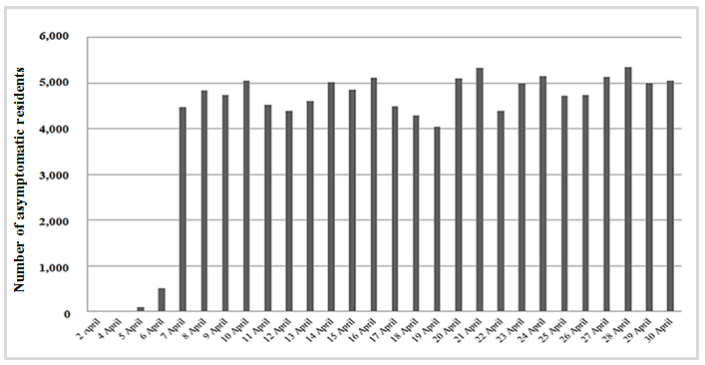
Numbers of residents who were asymptomatic for coronavirus disease for more than 14 days as reported by long-term care facility health care staff through COVIDApp over 30 days.

In contrast, the number of confirmed COVID-19 individuals increased progressively until day 22 and remained stable during the last week. Over the 30 days, the number of residents asymptomatic for more than 14 days was stable (5,090 of 10,347 (49.2%), Figure 2).

**Figure 3:**
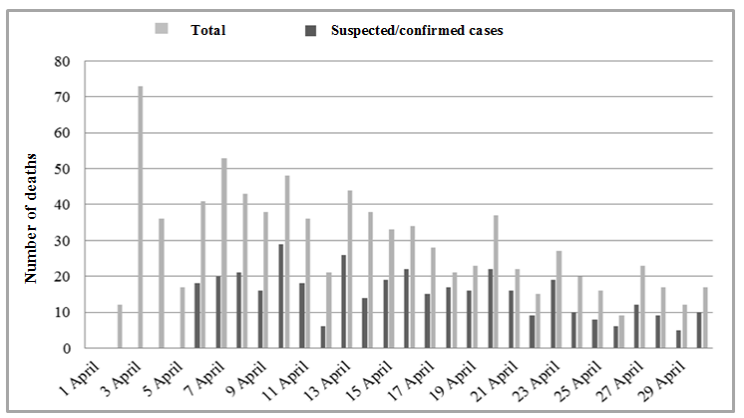
Total number of deaths and deaths in suspected/confirmed cases among residents, as reported by LTCF health care staff over 30 days.

Long-term care facilities reported a total of 854/10,347 (8.3%) institutionalized deaths during the 30 days; of these, 383 (44.8%) were suspected/confirmed cases. Figure 3 shows the progress of the deaths over the 30 days; increases were observed in both the total number of deaths and the deaths among suspected/confirmed cases during the first 2 weeks, followed by a progressive decrease. This decrease was more marked from the third week onward.

**Figure 4:**
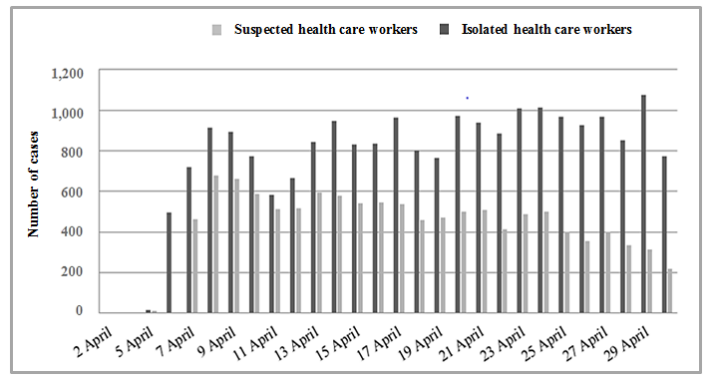
Number of suspected cases in health care workers and number of isolated health care workers, as reported by LTCF healthcare staff over 30 days.

Figure 4 shows the progress of suspected cases and isolated cases by center among health care staff working in long-term care facilities. The number of isolated health care workers (suspected or confirmed cases or contact with a confirmed case) remained high over the 30 days, although the number of suspected cases decreased during the last 2 weeks; this decrease became more apparent during the last week.

The number of long-term care facilities considered to be high-risk for COVID-19 decreased progressively from 19/196 (9.7%) to 3/196 (1.5%).

**Table 1:** Weekly data on the number of institutionalized residents, percentages of centers that reported data on the COVIDApp platform, and number of facilities considered to be high-risk.

Week (2020)	Residents, n	Centers that reported data, n (%)	High-risk centers, n (%)
April 9	10,347	174 (88.8)	19 (9.7)
April 16	10,089	177 (90.3)	8 (4.0)
April 23	9909	187 (95.4)	5 (2.5)
April 30	9785	190 (96.9)	3 (1.5)

## Discussion

### Principal Findings

Our app helped institutional staff from long-term care facilities and primary care clinicians address the COVID-19 pandemic by providing a new channel for real-time communication. The strategy was focused on 3 actions: first, early detection of suspected COVID-19 cases and rapid development of epidemiological actions such as self-isolation of suspected cases and contacts and relocation after positive or negative results; second, remote management of mild COVID-19 cases treated in institutions; and third, knowledge of progression of the infection in long-term care facilities (progress of confirmed cases, isolated and asymptomatic residents, number of isolated health care workers, and management of risk of spreading the infection in LTCF with a high number of risk factors for negative outcomes).

With data and guidelines still in development, health care professionals are fighting the COVID-19 pandemic on multiple fronts, and support tools are needed to manage the situation due to the complete saturation of national health systems (both primary and hospital care). In this context, telemedicine could be promoted for early diagnosis, patient isolation, and contact tracing. However, few data have been reported to date with respect to the use of technological platforms in the management of the COVID-19 pandemic [12-16]. Preliminary data indicates that telemedicine technologies, particularly video consultations, have been enhanced and scaled up to reduce the risk of transmission by monitoring symptomatic individuals in the United Kingdom [13] and the United States [14,15]. In France, Rolland et al [17] described the use of telemedicine to advise and support older people in nursing homes through a website that enables direct contact between a senior geriatrician and centers for older people. Much like our system, this approach enables diagnosis and monitoring of cases with COVID-19 in a care setting by mobile teams. However, no epidemiological data have yet become available from these studies.

Despite the limitations imposed by the COVID-19 pandemic, we were able to monitor the progress of the infection over 4 weeks of the pandemic in our area. We observed an initial gradual increase in the number of suspected and confirmed cases of COVID-19 with subsequent stabilization, together with a decrease in the number of deaths and an increase in the number of residents without symptoms for more than 14 days. Among health care workers, the number of suspected cases decreased during the last weeks of the study. COVIDApp enabled us to intervene proactively by isolating residents with suspected infection early and by monitoring contacts, not only among residents but also among health care workers, who are at high risk of COVID-19 infection. In this sense, the app proved to be a powerful tool for monitoring individuals living in health care institutions and the status of long-term care facilities and their health care workers during the COVID-19 pandemic. Consequently, the number of high-risk centers decreased during the study period. Monitoring centers at high risk of infection by detecting key risk factors appears to be essential if we are to minimize the spread of COVID-19 in long-term care facilities. The factors contributing to the vulnerability of these facilities were summarized by McMichael et al [18] as follows: working while symptomatic or working in more than one facility; inadequate familiarity with and adherence to standard, droplet, and contact precautions and eye protection recommendations; difficulty implementing infection control practices, including inadequate supplies of PPE and other items (eg, alcohol-based hand sanitizer); delayed recognition of cases because of a low index of suspicion, limited testing availability, and difficulty identifying persons with COVID-19 based on signs and symptoms alone.

Our strategy was based on detection and monitoring of suspected cases but also has a double epidemiological objective: to reduce transmission in a vulnerable population (residents and long-term care facility health care workers) and to monitor the progress of the infection in these centers [8,10,19]. Several authors have suggested that the consequences of insufficient response to epidemics in long-term care facilities could be severe in older persons, who are by definition frail and immunologically naïve to the virus [9,20].

### Limitations

Our tool was implemented in the midst of a pandemic, which necessarily implies a series of limitations. First, data must be interpreted with caution because they are reported and registered by long-term care facility staff for use in clinical care planning, although the data were validated by the primary care teams. Second, despite our conviction of the usefulness of the tool, implementation was difficult to consolidate due to the complexity of reporting the clinical status of individuals, especially in long-term care facilities experiencing multiple difficulties managing the crisis. In the near future, it will be necessary to work more closely with the staff of these facilities to improve individual reporting of signs, symptoms, and other clinical information as well as to introduce additional functionalities of the app. The need for rapid implementation of the app resulting from the urgency of the situation enabled us to manage COVID-19 in these centers; however, continuous changes in the platform have been necessary to ensure universal implementation and to optimize clinical data (monitoring of symptoms and vital signs and inclusion of additional clinical and epidemiological data). In addition, other aspects (eg, laboratory data, adherence to treatment, and adverse events) must be tested in future analyses under conditions of clinical practice.

### Conclusion

The COVID-19 pandemic has highlighted the need to optimize existing resources to prevent the collapse of health systems. COVIDApp is an innovative tool that can help clinicians rapidly detect and remotely monitor suspected and confirmed cases of COVID-19 in institutions, thus limiting the risk of spreading the virus. In addition, the platform shows the characteristics and progression of the situation in real time, thus facilitating the design of strategies tailored to a specific setting. Cost-benefit studies are necessary to measure the real benefits of such strategies.

## Abbreviations

COVID-19: coronavirus disease
PPE: personal protective equipment
RT-PCR: reverse transcriptase–polymerase chain reaction
SARS-CoV-2: severe acute respiratory syndrome coronavirus 2
TLS: transport layer security
